# Electric Shovel Teeth Missing Detection Method Based on Deep Learning

**DOI:** 10.1155/2021/6503029

**Published:** 2021-11-22

**Authors:** Xiaobo Liu, Xianglong Qi, Yiming Jiang

**Affiliations:** ^1^Shenyang Institute of Automation Chinese Academy of Sciences, Shenyang 110169, China; ^2^Liaoning Huading Technology Co.Ltd, Shenyang, Liaoning 110167, China; ^3^State Grid Liaoning Electric Power Company Limited Material Branch Company, Shenyang, China

## Abstract

Electric shovels are widely used in the mining industry to dig ore, and the teeth in shovels' bucket can be lost due to the tremendous pressure exerted by ore materials during operation. When the teeth fall off and enter the crusher with other ore materials, serious damages to crusher gears and other equipment happen, which causes millions of economic loss, because it is made of high-manganese steel. Thus, it is urgent to develop an efficient and automatic algorithm for detecting broken teeth. However, existing methods for detecting broken teeth have little effect and most research studies depended on sensor skills, which will be disturbed by closed cavity in shovel and not stable in practice. In this paper, we present an intelligent computer vision system for monitoring teeth condition and detecting missing teeth. Since the pixel-level algorithm is carried out, the amount of calculation should be reduced to improve the superiority of the algorithm. To release computational pressure of subsequent work, salient detection based on deep learning is proposed for extracting the key frame images from video flow taken by the camera installed on the shovel including the teeth we intend to analyze. Additionally, in order to more efficiently monitor teeth condition and detect missing teeth, semantic segmentation based on deep learning is processed to get the relative position of the teeth in the image. Once semantic segmentation is done, floating images containing the shape of teeth are obtained. Then, to detect missing teeth effectively, image registration is proposed. Finally, the result of image registration shows whether teeth are missing or not, and the system will immediately alert staff to check the shovel when teeth fall off. Through sufficient experiments, statistical result had demonstrated superiority of our presented model that serves more promising prospect in mining industry.

## 1. Introduction

Shovel excavators are widely used as primary production equipment in surface mining for removing overburden and ore materials due to their flexibility and mobility [1]. They dig the work front and load mining trucks in a three- or four-pass loading cycle. The shovel consists of three major assemblies: lower frame, upper frame, and attachment bucket [2], which is responsible for digging up the payload to the trucks. The bucket tooth of the loader is an important component of the loader structure [3], as well as a vulnerable part; the mounting part of the bucket tooth is at the front end of the bucket in order to reduce friction [4]. In the process of shoveling loading, the bucket tooth is in contact with the rock material directly, so its working condition is very bad; it not only increases the wear amount of the bucket tooth but also causes the teeth of the bucket to break and fall off. After the tooth is broken, it will cause a series of troubles, such as increasing the frictional resistance of the shovel during the loading operation, accelerating the wear of the bucket, and affecting its service life. The tooth must be replaced in time if it breaks. According to statistics, there are currently 2,000 to 2,500 large-scale mining excavators in service in China, and the annual direct economic loss due to the failure of the tooth reaches approximately 30 million yuan. What is even worse is that the teeth made of high-manganese steel have much higher hardness than the ore material. If the hard tooth enters the crusher along with the ore material, serious damage to the crusher gears and the production line is expected. At present, it is impossible to completely avoid the fracture of the tooth although many scholars have carried out a lot of research work from the aspects of the tooth mechanical analysis [5], the optimum replacement time for shovel teeth [6], the tooth material [7], and the heat treatment method [8].

In this case, machine vision technology combined with an automatic detection algorithm can monitor the working state of the tooth in real-time. When the tooth is falling off, it can provide accurate warning information and instruct the staff to deal with it in time, which has important practical significance and economic value for the production of the mine. Many scholars and enterprises have done a lot of research work on the detection of the shovel teeth breaking and falling off. Xiujuan Luo proposed a detection system solution, which uses a laser range finder to scan and reconstruct the three-dimensional model of the tooth in the working state and then compare it with the original model to determine whether there is any tooth drop in 2004 [9]. To improve the calculation speed, it uses the parallel computing method to collect and process the data synchronously so that the whole detection system has a better real-time performance. This method is less affected by changes in illumination and environment and has a good detection effect compared with machine vision detection methods. He Li proposed a novel object detection algorithm with the combination of appearance, structural, and shape features in 2011 [10]. It follows the paradigm of object detection by parts, which first uses detectors developed for individual parts of an object and then imposes structural constraints among the parts for the detection of the entire object. They further incorporate a priori shape information about the object parts for their detection, to improve the performance of the object detector. For validation of this object detection algorithm, they apply it to the detection of the tooth line of a mining shovel, under various lighting conditions. The experimental results demonstrate that this system can improve the detection performance significantly when part shape information is used. Ser Nam Lim presented a vision system for monitoring tooth condition and detecting missing tooth for a mining shovel in 2016 [11]. First, it extracts the image sample of the bucket during the movement of the shovel and uses this sample information to locate the approximate position of the tooth. Second, it uses the frame difference method and the optical flow method to correct the result and then uses the template matching and the tooth line fitting method to accurately locate the tooth target. Finally, it combines the grayscale features of the tooth image to determine if the tooth is falling off. The outstanding performance and high reliability of the proposed system have been demonstrated by experiments on video sequences collected from an iron ore mining site and a two-month trial of an installed unit on a production line. Abdallah Alma'aitah utilized Sprouts WSN platform to monitor the steel shovel teeth on shoveling equipment used in oil sands mining operations in Ft. McMurray, Alberta, Canada [12]. The Sprouts platform is utilized to monitor the viability of the shovel teeth on the shovel bucket's teeth adapter. Besides, if a shovel-tooth becomes detached, Sprouts is used to estimate its location. Hannington focuses on a method to prevent the teeth from ending up in the crusher based on an infrared detection system that reads the bucket profile every time it hauls the ore onto the truck in 2008. At the same time, another detection system available on the market has been studied and a probable preventive maintenance program has been suggested [13].

With the rapid development of deep learning skills in multiple areas [14–17], in this paper, a novel electric shovel teeth missing detection method which includes the following significant steps is proposed to monitor teeth condition and detect missing teeth. At first, salient object detection based on deep learning is presented for extracting crucial frames in video flow efficiently and releasing computational pressure of the terminal end. Additionally, the working environment of shovel teeth is relatively special, and the messy ore will produce a lot of noise in the frame images. Semantic segmentation is proposed to get the relative position of teeth in a noisy background. Finally, to detect the broken teeth, an image registration approach is introduced. With the proposed procedure, without human being affected, the electric shovel teeth missing detection method is ensured automatically and results in a high-accuracy rate. Our proposed procedure for missing tooth detection serves a fully automatic quality, which provides great significance in mining industry. If this work is supported by human resources, it should increase dangerous risks especially in low visible environments. Moreover, depending on real-time human supervised detection, the accuracy cannot be ensured and will result in great costs from economic perspectives.

Our main contributions are summarized as follows:

1. A salient object detection based on deep learning is proposed for deleting useless frame images and releasing computational pressure of subsequent work. Because salient detection is completed at the edge, the efficiency of the pixel-level algorithm is improved.
2. Semantic segmentation can find the relative position of teeth in the frame images and solve the noise problem generated by the working environment of the electric shovel. Compared with the previous method based on machine vision, the experimental result of semantic segmentation is more effective.
3. Image registration converts teeth into rectangular structures; by processing the results of the image registration experiment, the proposed strategy is capable of handling the system uncertainties caused by the working environment of electric shovel teeth. The final results show that the electric shovel teeth detection algorithm works with high accuracy in actual production.

## 2. Methodology

The proposed method is an intelligent computer vision system, which consists of salient object detection, image segmentation, and image registration. Generally, the number of shovel teeth (*M*) is fixed, *M*=5. This invariant value can be taken as an indicator of the detection of missing teeth and key frame extraction. By setting the number of detected teeth as *S*, we adapt the following formula to determine whether the teeth are missing or not:

(1) $$\begin{array}{c} \left\{\begin{array}{c} S=0\\ 0<S<M.\\ S=M \end{array}\right.. \end{array}$$

When *S*=0, there is no tooth in the image; when 0 < *S* < *M*, the teeth are broken; when *S*=*M*, it is in a normal state.

### 2.1. Salient Object Detection

**Figure 1:**
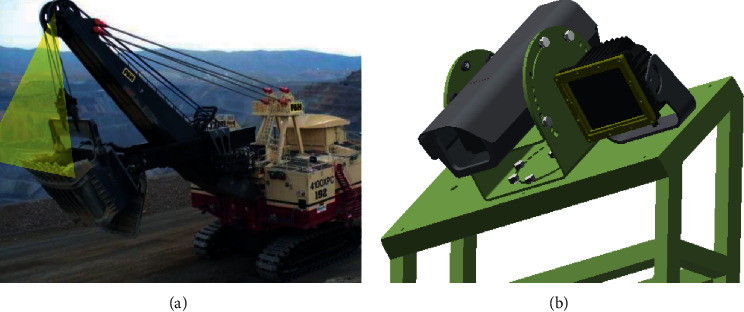
Illustration of our proposed solution on automatic electric shovel teeth detection.

In the mining process, dozens of mining machines are working at the same time. To more efficiently detect whether the tooth is missing on the shovel in each machine, a camera is installed on each shovel to monitor the status of the teeth. The position of the camera is significant for the detected system. The best position should allow the mounted camera to be stable during operation as shown in Figure 1. In the process of monitoring the state of teeth, the frame images are extracted from the video frame obtained by the camera for edge calculation. If every frame image in the video stream is sent to the server terminal, the computation will be very large, which will reduce the computational efficiency and require highly on the service facilities.

To alleviate the pressure of the data processing center and ensure the cost of facilities, we proposed a strategy based on salient object detection to obtain more significant frames in video flow and delete unnecessary frames. The purpose of this paper to detect whether the tooth on the shovel is missing or not and give a timely alarm when the tooth is missing. Therefore, when there is no tooth in the frame image, we can identify that this frame image is an unnecessary frame image. The function of salient object detection is to find the region of interest (ROI), and obviously, in this paper, our ROI is the teeth. The idea of salient object detection in deep learning depends on pixel values in feature maps located in deeper layers of neural networks. In general, pixels with great values indicate the salient section of the foreground we explore.

However, traditional salient object detection based on deep learning has many problems:

- The training is divided into several stages, and the steps are relatively complicated: fine-tuning network + training SVM + training border regression
- The training is time-consuming and takes up a lot of disk space: 5,000 images produce hundreds of gigabytes of feature files
- Computing slowly: it takes 47s for VGG-16 to process an image by using GPU

**Figure 2:**
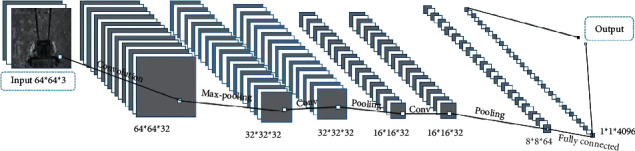
The structure of classification network for salient detection.

Low computational efficiency and a large amount of computation are not what we are willing to see in edge calculation. Based on these shortcomings of traditional salient object detection and our purpose to delete unnecessary frame images, a neural network of dichotomy is proposed, retaining frame images labeled with teeth and deleting frame images with no teeth. The network structure is shown in Figure 2.

The model contains three simple convolutional layers and two full connection layers. By calculating the parameters in this network, it can be found that the complexity of the network structure is lower than that of AlexNet. The low complexity network structure determines the efficiency of edge computation and improves the efficiency of the whole system under the premise of realizing salient detection.

### 2.2. Image Segmentation

After the salient object detection, the key frame images are obtained. To facilitate the subsequent image registration to detect the shovel teeth, image segmentation is carried out to extract the information of teeth. In the process of salient object detection, it is noticed that the position of teeth is relatively skewed, and the size of the teeth is too small. Therefore, the common convolutional neural networks are not able to complete high-accuracy image segmentation because of inability to obtain information about teeth. In this case, the DeepLabV3+ model is adopted to realize accurate acquisition of teeth information by using dilated convolution and finally to process image registration to realize missing teeth detection. To guarantee the scientific nature of the experiment, the dilation rate is changed to zero as a common convolutional neural network for the contrast test. By contrasting the result of the normal convolution neural network and the result of the dilated convolution neural network, it shows that the dilated convolution is more effective.

**Figure 3:**
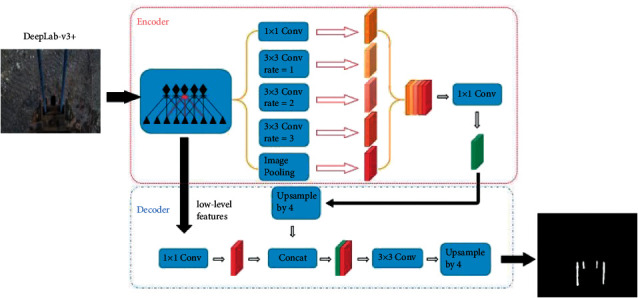
DeepLabV3+ structure.

DeepLabV3+ is an efficient model for image segmentation. It extends DeepLabV3+ optimization results with a simple and efficient decoder module, especially along the target boundaries. Besides, in the encoder-decoder structure, the resolution of the extracted encoder features can be controlled arbitrarily using dilated convolution to compromise accuracy and running time. Atrous Spatial Pyramid Pooling (ASPP) module contains four dilated convolutional layers and one global pooling layer. In the experiment of this paper, more efficient image segmentation is achieved by adjusting the dilation rate of the convolution kernel in ASPP. Decoder carries out 1 × 1 convolution to reduce channels before the fusion of low-level information. Then, the upsampling of the encoder's results is quadrupled and combined with the previous convolution and 3 × 3 convolution is carried out. Finally, the final result is obtained by quadrupling the upsampling. The specific network structure is shown in Figure 3.

To obtain more pixel information, under the permission of no adding parameters, it contains dilated convolutional layers in DeepLabV3+. However, there is the gridding effect [18] in the dilated convolution since the convolution kernels are not continuous in deep convolutional layers. In this case, there are always some pixels not involved in the calculation. Moreover, it is difficult to realize the segmentation of small objects in the dilated convolution with a large dilation rate. Since the relative size of teeth in this paper is small in the image, the dilated convolution with a small dilation rate is proposed to ensure the accurate segmentation of the teeth. To ensure that all pixels are involved in the calculation, the dilation rates in the deep convolutional layers satisfy the following formula:

(2) $$\begin{array}{c} M_{i}=\max\left[M\left(i1\right)-2r\left(i1\right)M\left(i1\right)-2\left(M_{i}1-r_{i}\right),r_{i}\right], \end{array}$$

where *M*_*i*_ is the maximum dilation rate of the *i*th layer and *r*_*i*_ denotes the dilation rate of the *i*th layer, with *M*_*n*_=*r*_*n*_.

In the actual work of detection, the relative position and size of the teeth in the image recorded by the camera are constantly changing, which is a challenging difficulty. DeepLabV3+ adopts a combination of dilated convolution cascade and parallel with multiple dilation rates to effectively extract multiscale semantic information. As a result, no matter where the relative position of the teeth and no matter what the size of teeth, the model processes image segmentation efficiently. Also, by combining image-level information, the ability of ASPP to extract global semantic information is improved to further improve the effect.

### 2.3. Image Registration

**Figure 4:**
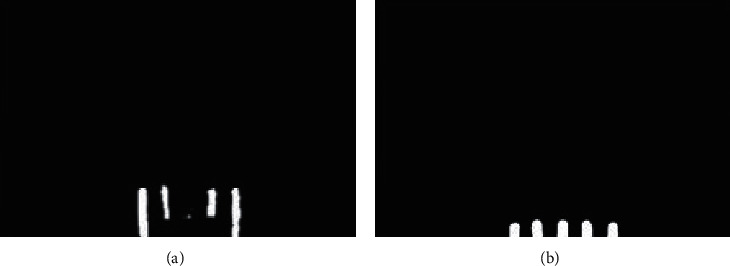
Segmented images.

After the image segmentation, binary images containing only teeth are obtained, as shown in Figure 4(a). In this section, the image registration is proposed to detect whether the teeth are missing or not. The image in which the number of teeth (*M*) is zero is deleted in the salient object detection. Therefore, in the process of image registration, the number of teeth (*M*) ranges from one to five.

In the process of image registration, the reference image is set up firstly. In this paper, the images obtained after image segmentation are highly similar, except the relative position of teeth is different. Also, the relative position of each tooth is the same. Therefore, one relatively clear image obtained from image segmentation with intact teeth can be used as the reference image. The final reference image shows five nearly identical rectangular structures at the bottom of the binary image, as shown in Figure 4(b).

**Figure 5:**
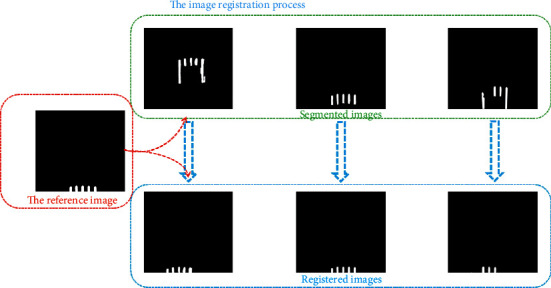
The image registration process.

However, in the segmented images, the relative position of teeth is different, and the pixels' information of the images is also very different. The reference image and the process of image registration are shown in Figure 4. As shown in Figure 5, the teeth in registered images are at the bottom of the image and have obvious rectangular structures. When there is no tooth missing, the registered image contains five rectangular structures. When the teeth are missing, the number of the rectangular structure is less than five. Moreover, the registered image is a binary image, which is very beneficial to some processing methods such as edge detection. Therefore, we detect the rectangles in the registered image and calculate the number of rectangles to determine whether the teeth are missing or not.

## 3. Experiments and Discussion

The experiments that we conducted are divided into three main sections: (1) salient object detection, (2) image segmentation, and (3) image registration.

The first sets of experiments are conducted to delete images without teeth to reduce the amount of computation, and the second sets of experiments are conducted to find the region of interest (ROI) and the location of teeth in the images. Moreover, it should be noticed that multiple dilation rates are proposed to extract multisemantic information, and the dilated convolution neural network is contracted to a normal convolution to demonstrate that the DeepLabV3+ model works more effectively. Third, image registration is conducted to detect whether the teeth are missing or not by counting the number of rectangles.

### 3.1. Datasets and Hardware

The datasets in this paper are from frame images of the video stream taken by the camera on the shovel. Frame images are extracted from the video stream, consisting of 500 with and 500 without teeth. After labeling these images, twenty percent of the images are used as a validation set to test the training model, and the other eighty percent of the images are used as the training set to train the classification model. The training and inference of our proposed model are run with two NVIDIA GTX1080 Ti 11 GB GPUs, 32G RAM, and Intel i7-7700K CPU with 8 cores 4.20 GHz. Especially, we did not adopt any preprocessing of noise management for our dataset in order to preserve original details and avoid possible artifacts for training and inference process.

### 3.2. Salient Object Detection Studies

To reduce the computation of the terminal server, salient object detection is proposed to delete the images without teeth. To solve the shortcomings of traditional salient object detection, a dichotomous neural network is adapted to delete the image labeled no teeth.

#### 3.2.1. Preprocessing

This set of experiments aims to use edge computing to alleviate the pressure of the terminal server. Therefore, to complete the edge computing faster and reduce the calculation amount, the images are resized into 64 ×64× 3.

#### 3.2.2. Parameters

The network's detailed connectivity and kernel configuration are illustrated in Figure 2. And all the convolutional kernels' sizes are the same as the size of 3 × 3. The pooling layer of every convolutional layer is MaxPooling, the kernel size is 2 × 2, and the step size is 2. The number of this neural network is 233,472, which is less than half of the AlexNet. Adam optimizer is adapted with a learning rate of 0.0001. The batch size is set as 32, and the number of iterations is set as 8,000.

#### 3.2.3. Training Process

**Figure 6:**
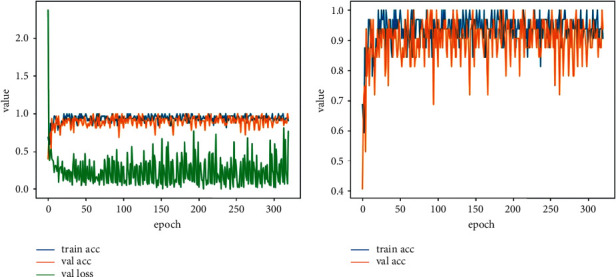
Training metrics.

Normally, too many iterations may cause the model to fit the noise and unnecessary features of the training sets, and then overfitting occurs. In the case of overfitting, the model is of high complexity and does not work to the data except the training set. To avoid excessive computation, the loss function of the validation dataset and the accuracy of the validation dataset are adapted as the evaluation metrics for model selection. When the accuracy of the validation dataset reaches a high level and the accuracy is not improved after 10 epochs, also the loss function is not declined, the iteration is terminating in advance. The final model is obtained. In the training process, the accuracy of the training dataset, the accuracy of the validation dataset, and the loss function of the validation dataset are shown in Figure 6.

**Figure 7:**
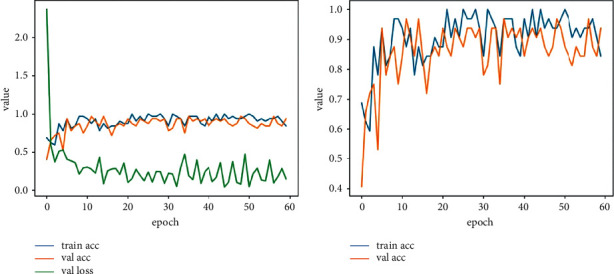
Training metrics (less iterations).

As shown in Figure 6, the accuracy of the validation dataset and the training dataset reaches the global maximum within fifty epochs, and the loss function of the validation dataset does not decrease in the subsequent epochs. As shown in Figure 6, the accuracy of training and validation fluctuates periodically since some groups of sampled images contain lots of noise as a result of complicated wild working environments, which leads to poor performances of semantic segmentation. Therefore, to prevent the training model from overfitting, the number of iterations is changed into 1,600 making the epochs to 64, reducing the training time and calculation amount of the training model. The training result is shown in Figure 7.

## 4. Results

As can be seen from Figure 7, in the 56th epoch, the accuracy of the training dataset and the accuracy and the loss function of the validation dataset reach the optimal level. The accuracy of the training dataset is 93.75%, the accuracy of the validation dataset is 96.87%, and the loss function of the validation is 0.0965. Therefore, “teeth-no teeth-model-1400” generated by the model trained to the 56th epoch is adapted to detect whether the teeth are missing or not.

One video stream of a working shovel is incepted, all frame images are extracted from it, and the frame images are put into the trained model for prediction. If the prediction result of the image is “teeth,” this image is kept for the next part of the experiments. Otherwise, this image is deleted. In the prediction experiment of the frame images of the captured video stream, the prediction results are all correct, indicating that the model predicts effectively. In the three convolutional layers of the network, the feature maps of images with teeth and without teeth are shown in the following figures. It is through learning these features that our model can correctly predict whether the images contain teeth or not and then complete the salient object detection.

### 4.1. Image Segmentation

To detect whether the teeth are missing or not, the relative position of the teeth should be found at first. The DeepLabV3+ model is adapted for processing semantic segmentation to find the location of the teeth. The dilation rate is adjusted several times in our model to avoid missing information in the convolution process. Then, to verify the advantages of dilated convolution, the dilation rate in the network is changed to zero for a comparison experiment.

#### 4.1.1. Preprocess

**Figure 8:**
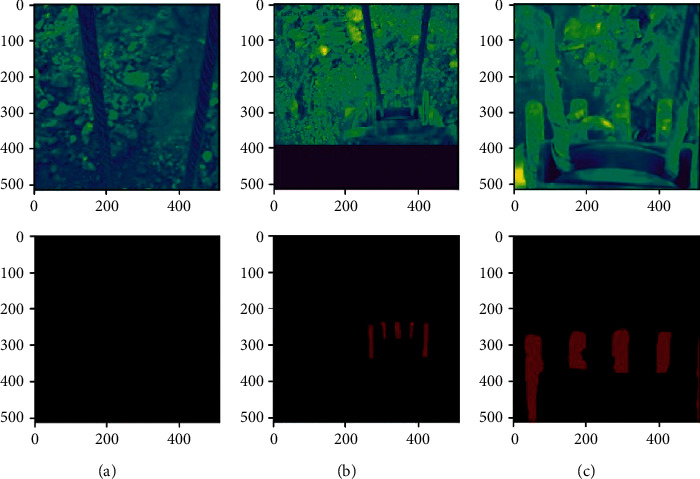
Labeled images.

First, to facilitate the later training process, the frame images are resized as 512 × 512. Then, the region of interest of the images is annotated through the “labelme,” an image labeling tool. Images with teeth are labeled with the location of teeth, and the images without teeth do not need any operation. After the image labeling is completed, to ensure the rationality of our model, the image is processed by methods such as rotation, scaling, and cropping, and the processed image is added to the data as new images. The labeled image is shown in Figure 8.

#### 4.1.2. Parameters

The details of the DeepLabV3+ are shown in Figure 3. In this section, the structure of the model will not be changed, but the dilation rates are changed to prevent the information missing from the dilated convolution. To obtain the information of each pixel by dilated convolution, the dilation rates are changed to 0, 1, 2, and 3. Later, to prove the superiority of the dilated convolution for image segmentation, the dilated rates are set as 0, 0, 0, and 0 for comparison experiments.

#### 4.1.3. Training

The colorful images are transformed into grayscale images for training, reducing the color difference noise brought by the day and night. The initial learning rate is set as 0.01, and the learning rate will be adjusted dynamically [19].

#### 4.1.4. Evaluation Metrics

(i) Pixel accuracy (PA) means the ratio of correctly classified pixel points in all pixel points. In this paper, there are only two kinds of classes of the pixel: teeth and background. *P*_10_ represents the total number of pixels in the background predicted to be in the teeth. *P*_11_ represents true positive, *P*_10_ means false positives, *P*_01_ means false negative, and *P*_00_ represents true negative:
  (3) $$\begin{array}{c} \text{PA}=\frac{P_{11}+P_{00}}{P_{11}+P_{10}+P_{01}+P_{00}}. \end{array}$$
(ii) Mean intersection over union (MIOU) is the standard metric of semantic segmentation. It calculates the ratio of the intersection and union of two sets, the real value, and the predicted value. Calculating the intersection over union for each class and averaging it, then the MIOU is obtained as follows:

(4) $$\begin{array}{c} \text{MIOU}=\frac{1}{2}\left(\frac{P_{11}}{P_{11}+P_{10}+P_{01}}+\frac{P_{00}}{P_{01}+P_{00}+P_{10}}\right). \end{array}$$

#### 4.1.5. Result

**Figure 9:**
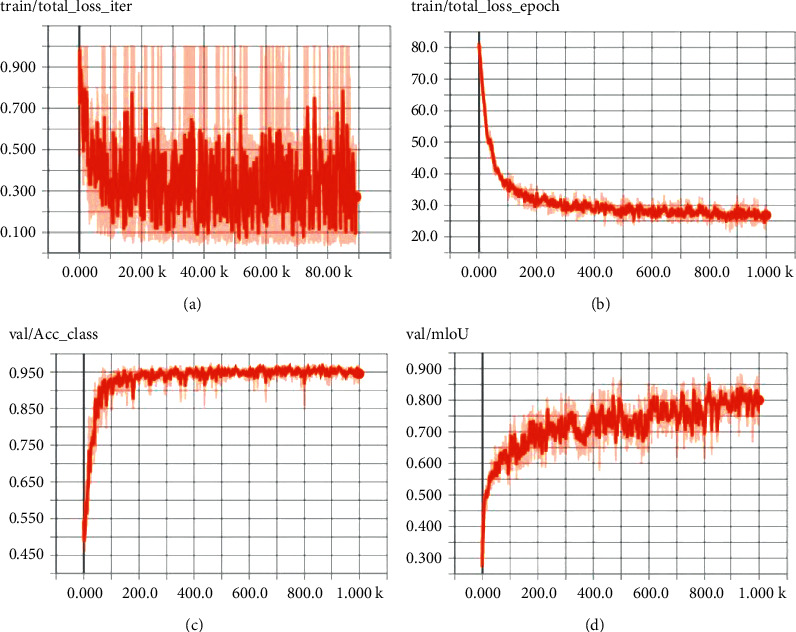
Evaluation metrics: (a) loss function in one iteration; (b) loss function in epoch; (c) PA; (d) MIOU.

After about 1,000 epochs of training, a relatively efficient semantic segmentation model for teeth is obtained. The variation trend of the loss function, pixel accuracy rate, and MIOU in the training process is shown in Figure 9.

**Figure 10:**
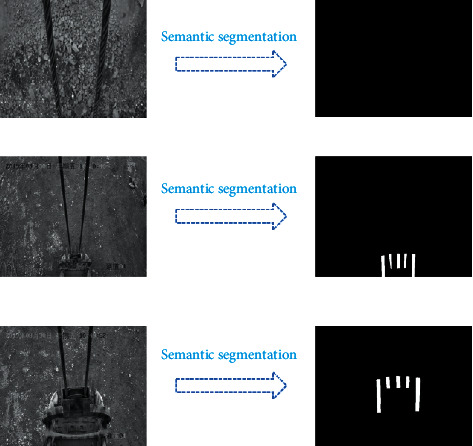
The segmented images.

As can be seen from Figure 9, after 1,000 times of training, all the indicators have stabilized. However, to avoid overfitting caused by too many iterations, the training result of the 512th epoch is adapted as our model for semantic segmentation. It can be seen from Figure 9 that when the iterations reach the 512th epoch, the loss function in one epoch is stable at about 25, the loss function in one iteration is stable at about 0.3, PA is about 0.95, and MIOU is about 0.75. Thus, the model of the 512th epoch is a very efficient training result. The model is adapted to process dozens of images that are not in the training dataset to carry out semantic segmentation and find out the teeth in these images. Later, verifying the predicted results manually finds that the predicted results of the teeth are accurate. Moreover, the training dataset consists of grayscale images in the experiment, and the images of day and night could be accurately segmented out the teeth. The segmented results are shown in Figure 10.

#### 4.1.6. Comparative Experiments

**Figure 11:**
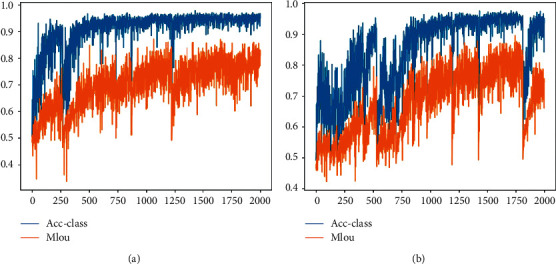
Evaluation metrics: (a) dilation convolution; (b) comparative experiment.

A comparative experiment is conducted to verify the validity of the dilation convolution. The dilated rates of the dilation convolution layers are changed to zero, and a new model is carried out. By observing the evaluation metrics of the model, the comparative experimental results are obtained as shown in Figure 11.

As shown in Figure 11, the results of the dilation convolution are better. The model is stable at the 500th epoch, but the comparative experiment will not converge until the 1,000th epoch. Moreover, the experimental results of the dilation convolution are relatively stable, while the results of the other show great differences after each epoch. Therefore, this set of comparative experiments effectively illustrates the excellent features of fast converge and stable experimental results of dilation convolution in teeth semantic segmentation.

### 4.2. Image Registration

After the image segmentation, the binary images with teeth are obtained. The number of teeth in these binary images is considered as the metric to discharge whether the teeth are missing or not. Since the shape of the tooth is similar to a rectangle, the number of rectangles is calculated in the image after registration. If the number of teeth is less than five, the teeth are considered missing; otherwise, it is intact.

**Figure 12:**
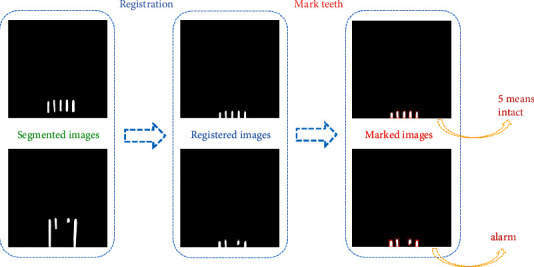
The registration result.

First of all, the reference image is a need in image registration. An image with relatively clear and intact teeth is selected as the reference image from the results of image registration. Because of the camera's angle of view, the teeth are always centered in the images and only move up or down. The registration process is not complicated. The objects of the image registration experiments are all the binary images with teeth obtained from image segmentation in previous experiments. After the image registration, the number of rectangles at the bottom of the images is calculated. Part of the experimental results is shown in Figure 12.

**Figure 13:**
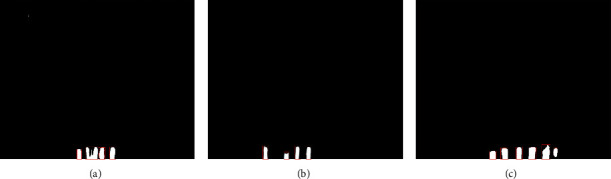
Special circumstances: (a) connected teeth; (b) broken teeth; (c) more teeth.

According to the number of rectangular structures in the registration experimental results, there are some defects in judging whether the teeth are missing or not. The rectangular structure is the smallest rectangle that covers each connected domain in the image. When multiple teeth are connected due to noise, as shown in Figure 13(a), the method of counting the number of rectangles fails. Besides, when the teeth are broken not fall off, as shown in Figure 13(b), the broken teeth will also have a rectangular structure after image registration. Due to noise, there may be more rectangular structures than the teeth in the result of image registration, as shown in Figure 13(c). As a result, this method does not work.

**Figure 14:**
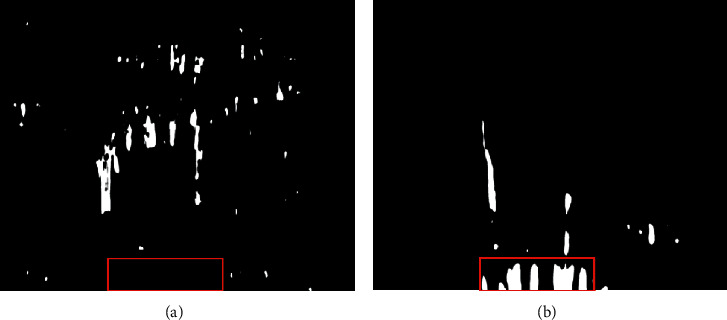
Registration abnormalities: (a) registered anomaly; (b) excessive noise.

Therefore, another method is proposed, which adapted the area ratio (*α*) of the teeth in a registered image to the reference image as the evaluation metric. As noise will affect the area, to more accurately judge whether the teeth are broken or fall off, the area of teeth in the registered image is calculated where the teeth are located in the reference image. In the image registration, due to noise, registration abnormalities will occur. As shown in Figure 14, the ratio of teeth area in the registered image to the reference image is too small or too large.

Without considering simultaneous falling off or fracture of multiple teeth, the following threshold values for judging whether teeth fall off or fracture can be obtained:

- *α* ≤ 0.6, registration anomaly
- 0.6 < *α* ≤ 0.8, a tooth falls off at least
- 0.8 < *α* ≤ 0.9, a tooth fractures
- 0.9 < *α* ≤ 1.1, teeth intact
- 1.1 < *α*, registration anomaly

To verify the effectiveness of the image registration, we conducted two groups of test experiments and observe the test results. The dataset of the two groups of experiments consists of the following: one is 70 positive samples without teeth missing and 35 negative samples with missing teeth and the other one is 65 positive samples and 20 negative samples. The test experiment results are shown in Table 1.

As can be seen from Table 1, all negative samples can be detected accurately, and the detection accuracy is more than 95%. However, there are still some images without broken teeth detected as broken teeth images, and there is still a false alarm rate, but it can still meet the missing detection function of teeth.

**Figure 15:**
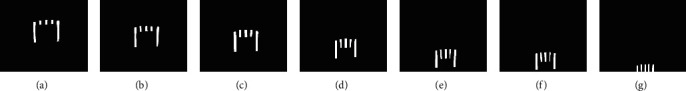
Denominator images.

By observing the images of false positives, the spatial position of the shovel is the culprit. When the shovel is far away from the camera, the relative size in the image is small. On the other hand, when the shovel is close to the camera, the relative size is large. Adapting multiple images is an efficient solution to handle this. These images are named”Denominator.” According to the differences in the spatial position of the shovel, seven reference images are selected, as shown in Figure 15.

**Figure 16:**
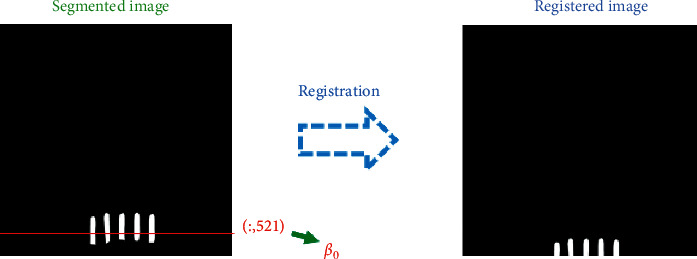
The coordinates of segmented images.

**Figure 17:**
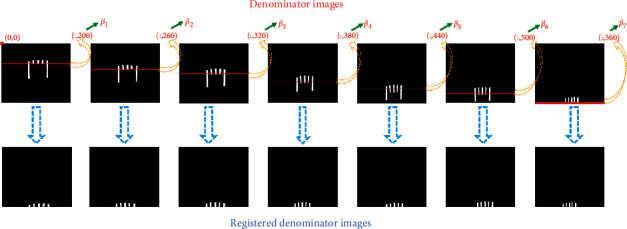
The coordinates of denominator images.

After the image registration, because rigid registration is adapted in this paper, the starting point of registration can be found by taking the pixel feature. As shown in Figure 16, the red line represents the starting point of image registration and obtains the ordinate of the red line(*β*_0_). From Figure 17, each denominator image has a registration starting point, which is *β*_1_, *β*_2_,…, *β*_7_. While *β*_*i*_ − 30 < *β*_0_ ≤ *β*(*i*+1) − 30, the *ith* denominator image is adapted in area ratio.

To show the experimental result more clearly, images with an area ratio less than 0.6 or large than 1.1 are deleted as abnormal registered images. The detection results can be divided into two categories:

- 0.6 < *α* ≤ threshold means abnormal teeth
- Threshold < *α* ≤ 1.1 means intact teeth

Meanwhile, the detection experiment is transformed into a two-classifier, and the selection of threshold is crucial. The ROC curve is adapted to obtain the best threshold.

After setting a range of threshold values, a classification table (confusion matrix) is built for each threshold value. Each table produces a point in the ROC curve [20].  Table 2 shows how the performances of these points are calculated in this study. These thresholds separate the dataset into two subsets: the error and no-error sets. As shown in the following formula, the coordinates of the points on the ROC curve are obtained from the confusion matrix:

(5) $$\begin{array}{c} \text{TPR}=\frac{\text{TP}}{\text{TP}+\text{FN}},\\ \text{FPR}=\frac{\text{FN}}{\text{TP}+\text{FN}}. \end{array}$$

**Figure 18:**
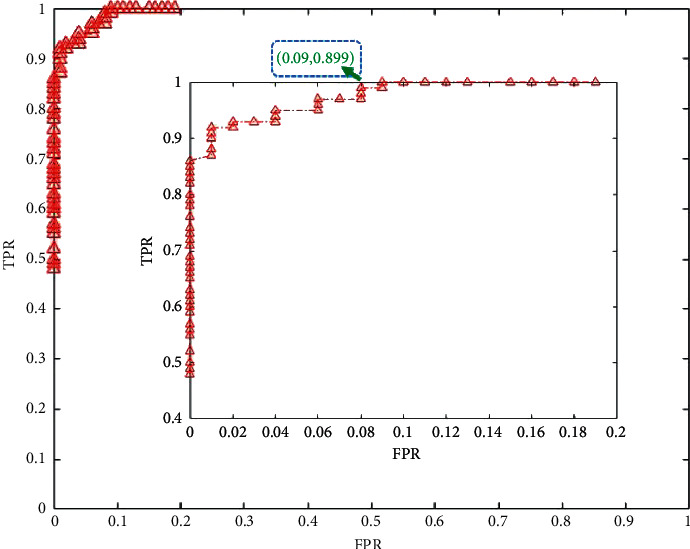
The ROC curve.

The closest point to point (0,1) is the best threshold of this classifier [21]. As shown in Figure 18, the coordinate of the closest point to point (0,1) is (0.09, 0.99) and the threshold of this point is 0.899. After selecting the corresponding denominator image, the threshold value at this time is close to the ideal value of 0.9, which shows the logical correctness of the experiment. The experiment in this paper has also been proved to have high working efficiency when it is applied in the mine of Anshan Iron and Steel Mining Company.

## 5. Conclusion

In this paper, a detection method based on deep learning is proposed for the detection of missing teeth of mine electric shovel. This method effectively solves the instability of the traditional method, can monitor the state of the shovel teeth in real-time, and can detect the teeth falling off or breaking in time. It has great practical significance for mine production.

## Figures and Tables

**Table 1:** Test result.

Positive samples	Negative samples	True positive	True negative	False positive
70	35	68	35	2
65	20	64	20	1

**Table 2:** Confusion matrix.

Classified	Actual	
	Abnormal teeth	Intact teeth
*α* ≤ threshold	True positive	False positive
*α* > threshold	False negative	True negative

## Data Availability

The raw data supporting the conclusions of this article will be made available by the authors, without undue reservation.
